# The 24-Form Tai Chi Improves Anxiety and Depression and Upregulates miR-17-92 in Coronary Heart Disease Patients After Percutaneous Coronary Intervention

**DOI:** 10.3389/fphys.2020.00149

**Published:** 2020-03-11

**Authors:** Jia Liu, Ping Yu, Wei Lv, Xinxin Wang

**Affiliations:** ^1^Department of Cardiovascular Medicine, The First Hospital of Jilin University, Changchun, China; ^2^Department of Cadre Ward, The First Hospital of Jilin University, Changchun, China

**Keywords:** 24-form Tai Chi, anxiety, depression, SF-36, coronary heart disease, percutaneous coronary intervention, miR-17-92

## Abstract

**Background:**

Anxiety and depression are common symptoms in patients with coronary heart disease (CHD) after percutaneous coronary intervention (PCI). The 24-form Tai Chi may exert a protective function for CHD patients after PCI by improving anxiety and depression.

**Methods:**

Patients who received PCI after 1–4 days were randomly assigned to the 24-form Tai Chi group (TG) and the control group (CG). The differences in anxiety and depression, using the Medical Outcomes Study 36−item Short−Form Health Survey (SF-36), before and after an average of 10 months of Tai Chi intervention were compared in both groups to analyze the effects of Tai Chi on the emotion and the life quality of CHD patients. Meanwhile, the relative levels of miR-17-92 were measured by using real-time qPCR. The association between the relative levels of miR-17-92 and the anxiety and the depression of CHD patients after PCI was analyzed. Adjusted Cox models were used to explore the effect of Tai Chi exercise in CHD patients.

**Results:**

After 10 months of intervention, the changes in the anxiety subscale (*P* = 0.002), in the depression subscale (*P* = 0.008), and in the stress (*P* = 0.015) scores were higher in the TG group when compared to those of the CG group. The proportion of anxious (*P* = 0.045) and depressed subjects (*P* = 0.042) in the TG group was lower than that in the CG group. On the other hand, the increase in the SF-36 scores and in the relative levels of miR-17-92 was significantly higher in the TG group when compared with that of the CG group (*P* < 0.05). The serum level of miR-17-92 had a negative correlation with the anxiety, the depression, and the stress scores (*P* < 0.01).

**Conclusion:**

The 24-form Tai Chi improved the anxiety and the depression symptoms and upregulated the miR-17-92 levels in CHD patients after PCI.

## Introduction

Coronary heart disease is a major disease that threatens human life and health and is one of the leading causes of death (Grabovac et al., 2018; Zhao et al., 2019). PCI is widely used in the treatment of CHD (Cheng et al., 2019; Dayoub et al., 2019). Although the cases of the patients with coronary artery restenosis and thrombosis can be reduced from 30–40 to 15% after 1 year of PCI, restenosis, and thrombosis are still serious clinical problems (Jaffery et al., 2011; Hofma et al., 2015). Therefore, controlling the risk factors of CHD is still necessary to prevent the occurrence of MACEs.

Depression (Hennessy et al., 2018; Monk et al., 2018; Zotcheva et al., 2019) and anxiety (Kasten et al., 2019; Ullmann et al., 2019; Zotcheva et al., 2019) are known indicators of poor outcomes in CHD (Palacios et al., 2018). Heart attack and PCI surgery also result in anxiety and depression (Zhang, 2015b). Anxiety and depression can produce multiple negative effects on patients by reducing their compliance with the treatment (Gehi et al., 2005; Bauer et al., 2012). Furthermore, the anxiety and the depression of CHD patients often occur together after PCI (Kala et al., 2016). There is still no effective way to control the symptoms of anxiety and depression.

Tai chi is a popular mind–body exercise in China that combines Chinese martial arts and meditative movements, and it can promote body balance (Adler et al., 2019), lessen depression, and improve cognitive function (Zhang et al., 2014; Sungkarat et al., 2018). The exercise needs mental concentration, physical balance, and muscle relaxation and shows a great potential in rehabilitating medical and psychological status (Yeh et al., 2009). Tai Chi even effectively treats adolescents with depression (Zhang et al., 2018). SSD and SSA are common in the elderly population and can cause suicide risk and disability. Tai Chi has been proven to improve SSD and SSA symptoms over 1 year of intervention (Rawtaer et al., 2015). Meta-analysis has shown that Tai Chi intervention reduced the Hamilton Depression Scale and Hamilton Anxiety Scale scores of stroke patients (Yang et al., 2018). Another meta-analysis has indicated that Tai Chi is a worthy complementary non-pharmacological resource which works by ameliorating depression and anxiety and may have great implications in public health (Zhang et al., 2019). However, the effects of Tai Chi on the anxiety and depression of patients with CHD after PCI and its related molecular mechanisms remain unclear.

miRNAs play important roles in the symptoms of depression (Ma et al., 2019) and anxiety (Du et al., 2019). miRNAs are non-coding RNA molecules with 18- to 28-bp nucleotides. They play critical roles in the posttranscriptional regulation of protein expression, which is involved with the normal and pathological cellular processes, including cell differentiation, cell cycle progression, and apoptosis (Tanase et al., 2012). miRNAs can affect the expression of various genes and may be the candidates associated with depression symptoms (Yuan et al., 2018). Tai Chi has been found to exert a protective function by affecting the serum levels of miRNA (Li et al., 2019) and may also improve depression and anxiety symptoms by affecting the miRNA.

The miR-17-92 cluster host gene spans 7 kb and contains the 800-nucleotide cluster transcript that encodes miR-17, miR-18a, miR-19a, miR-20a, miR-19b-1, and miR-92a-1. The miR-17-92 cluster is an important indicator in the development of the immune system, the heart, and the lung and in oncogenic patients. It is involved with cellular innate and adaptive immunity, such as B cells, T-lymphocyte subsets, T follicular helper cells, regulatory T cells, monocytes, macrophages, and so on (Kuo et al., 2019). The miR-17-92 cluster plays a critical role in cardiomyocyte proliferation (Gao et al., 2019) and affects the development of various diseases by regulating many related cellular processes and multiple target genes, including neurological diseases and cardiac diseases (Bai et al., 2019). The members of the miR-17-92 cluster and the expression of their passenger miRNAs expressions promote exercise-induced cardiac growth and reduce adverse ventricular remodeling (Shi et al., 2017). Cardiac rehabilitation will contribute in protecting against the development of depression and anxiety (Zheng et al., 2019). A recent study showed that miR-17-92 may regulate neurogenesis and anxiety- and depression-like behaviors (Jin et al., 2016). Tai Chi can affect the level of miRNA, and it possibly also affects the level of miR-17-92 because it can improve the symptoms of anxiety and depression (Rawtaer et al., 2015).

There are four major styles of Tai Chi, including Chen style (Zou et al., 2019), Yang style (also 24-form style) (Zou et al., 2017), Sun style (Liu and Cao, 2018), and Wu style (Taylor-Piliae et al., 2012). The former styles are effective in improving global cognitive function, balance, and fitness (Zou et al., 2019). However, Chen style is more complex than Yang style; thus, the latter style of Tai Chi (24-form) was tried in the present work. The present paper aims to investigate the effects of the 24-form Tai Chi on the anxiety and the depression of CHD patients after PCI.

## Materials and Methods

### Calculation of Sample Size

The sample size was calculated according to the following equation: *n* = (μ*_α_* + μ*_β_*)^2^ (1 + 1/*k*) σ^2^/δ^2^ according to a previous report (Nyklicek et al., 2014), where α = 0.05, β = 0.2, σ = 0.45, and δ = 0.38. δ stands for the smallest difference and δ = 0.38 will be detected when power is 0.8. The calculated sample size was 32. Considering 10% loss of follow-up, the sample size per group was 35.

### Inclusion Criteria

Before the present experiment, all processes were approved by the Human Research Ethics Committee of The First Hospital of Jilin University (Approval No. JLUFH-2468HD). The patients were included if they had one of the following criteria: (1) anxiety, depression, angina and dizziness, asthma, chills, sweating, nausea, and even syncope and other clinical symptoms; (2) coronary angiography showing that one of the three main coronary or left main porridge sclerosing lesions and luminal stenosis was more than 50%; (3) history of acute myocardial infarction and ECG showing old infarct Q waves; and (4) ST segment of ECG at rest or following exercise was horizontal or down-tilted lower than 1 mm, and continuous time was more than 2 min. PCI stent was implanted 1–4 days ago, and the patients agreed to sign an informed consent stating their willingness to participate in the study.

### Exclusion Criteria

The patients were excluded if they met one of following items: age over 70 years old and the treatment for mental illness and severe physical and psychological complications such as cancer, mental illness, and brain damage.

### General Questionnaire

The general questionnaire was created and modified according to a previous report (Akbaraly et al., 2015) and our demographic and clinical data. The general survey questionnaire included demographic data such as age, gender, height, weight, disease diagnosis, and PCI indication and clinical data such as the number of diseased blood vessels, stents, comorbidities, family history of cardiovascular disease, myocardial infarction, and medication being used. The anxiety scores were measured by using the General Anxiety Disorder 7-Item (GAD-7) (Johnson et al., 2019). The GAD-7 scores were classified as 0–4 (no anxiety symptoms), 5–9 (mild), 10–14 (moderate), and 15–21 (severe). Depression scores were measured by using the Patient Health Questionnaire-9 (PHQ-9) (Levis et al., 2019). The PHQ-9 scores were classified as 0–4 (no depressive symptoms), 5–9 (mild), 10–14 (moderate), 15–19 (moderately severe), and 20–27 (severe). All anxiety and depression questionnaires were asked on the Monday, Thursday, and Sunday within the week before intervention or prior to the end of intervention, and the mean scores were calculated.

### Hospital Anxiety and Depression Scale (Zigmond and Snaith, 1983)

The scale has 14 items, including sub-scales of anxiety and depression (seven items each), respectively. Points for each item are 0–3, and the highest scores for anxiety and depression are no more than 21 points, whereas the lowest score is 0. The anxiety subscales are 1, 3, 5, 7, 9, 11, and 13; the depression subscales are 2, 4, 6, 8, 10, 12, and 14. Among the anxiety subscales, the seventh item is scored in reverse, and the 2nd, 4th, 6th, 12th, and 14th items in the depression subscale are reversely scored. According to the original author’s criteria, the total score of the two subscales is 0–7, which means no anxiety or depression; a total score of 8–10 represents possible or critical anxiety or depression, and a total score of 11–21 points may indicate serious anxiety or depression.

### Perceived Stress Scale (Cohen et al., 1983)

The scale consists of 14 items, and each item has five points and reflects stress and sense of control. Seven out of the 14 items of PSS-14 are considered as negative (1, 2, 3, 8, 11, 12, and 14) and the remaining seven as positive (4, 5, 6, 7, 9, 10, and 13), representing perceived helplessness and self-efficacy, respectively. Each item was rated on a five-point Likert-type scale (0 = never to 4 = very often). The total scores are calculated after reversing the positive items’ scores and then summing up all scores. The total scores for PSS-14 range from 0 to 56. A higher score indicates greater stress. The scale was used to evaluate three stress scenarios: (1) daily chores, (2) major events, and (3) changes in stressors. The respondent answers the stress situation in the past month. The data showed good reliability and validity with the Cronbach’s α coefficient of 0.78. The correlation coefficient between the items was 0.28 on average, and the correlation coefficient between the total scores was 0.37–0.53. According to a previous report, Cronbach’s α coefficient of 0.78–0.88 indicates an adequate internal consistency (Jauregui-Lobera et al., 2011) and the inter-item correlation for the optimal level of homogeneity ranges from 0.2 to 0.4 (Briggs and Cheek, 1986), and so the present values showed high internal consistency and homogeneity.

### Measurement of Life Quality

The life quality of the CHD patients after PCI was measured by using the Medical Outcomes Study 36-item Short-Form Health Survey (SF-36) version 2, which consists of assessing PF (10 items), SF (two items), RP (four items), BP (two items), MH (five items), RE (three items), VT (four items), and GH (five items) (Ware, 2000).

### Patient Grouping

This is a single-blinded study, and informed consent was obtained from the patients who were eligible for the study within 1–4 days after PCI and who met the inclusion and exclusion criteria. Allocation concealment was performed to avoid subsequent interference. The enrolled patients were numbered according to the patient’s admission order and randomly divided into the 24-form Tai Chi group (TG) and the control group (CG) with equal allocation ratio by using a random number table generated by a computer. All CHD patients in the CG group received routine treatment, examination, nursing, and health education. The antidepressant amitriptyline was administered at a dose of 50–200 mg/day according to the different severity degrees of depression. The TG group received the same treatment as the CG group. Meanwhile, the 24-form Tai Chi exercise was provided with reference to the Health Qigong 24-form Taijiquan issued by the State Sports General Administration in 2003 and developed by recent work (Lee, 2017; Zou et al., 2017; Deng and Xia, 2018), performed two times per day starting from 6:00 to 8:00 am and from 16:00 to 17:00 pm at 50–60 min per session. According to the individual condition of the patient, the exercise intensity and the exercise volume should be adjusted according to the patient’s individual condition, and patient tolerance should be appropriate.

### Real-Time qPCR Analysis of miR-17-92

A sample of 5 ml of venous blood was taken from each subject under aseptic precautions, and the serum was prepared through centrifugation at 1,000 (×g for 10 min. All of the sera were collected at the time of diagnosis and the total RNA was extracted with the QIAamp Circulating Nucleic Acid Kit (Qiagen Inc., CA, United States). Reverse transcription-related operation was performed by using Qiagen’s QuantiTect Reverse Transcription Kit. The relative level of miR-17-92 was measured by using the SYBR Premix Ex Taq TM (TaKaRa, Dalian, China). The primers for miR-17-92 (forward primer 5′-TCATACACGTGGACCTAAC-3′ and reverse primer 5′- CTCTCTAAGAAACCAATCC-3′) and U6 (forward primer 5′-GCTTCGGCAGCACATATACTAAAAT-3′ and reverse primer 5′-CGCTTCACGAATTTGCGTGTCAT-3′) were synthesized by TaKaRa. The PCR was performed as follows: 95°C for 3 min, one cycle and 45 cycles of 20 s at 94°C and 40 s at 60°C, respectively. Default threshold settings were used as threshold cycle (Ct). After calculating the Ct value, the level of miR-17-92 was normalized to U6, and the relative quantification of miRNA expression was calculated with 2-ΔΔCt. The miRNA stability was measured by using the total RNA harvested at 4, 8, and 12 h (Mall et al., 2013), and the miRNA expression was confirmed using the abovementioned real-time qPCR.

### Statistical Analysis

Data analysis was performed using the SPSS 20.0 statistical software. The data were expressed as mean value ± SD (standard deviation). The count data were analyzed by chi-square test. The Kolmogorov–Smirnov (KS) and the Shapiro–Wilk tests were used to test the normal distribution of all variables (Nguyen et al., 2013). The two-sided *t*-test was used to compare the anxiety, depression, stress, and SF-36 scores before and after the test. The Mann–Whitney *U*-test was conducted instead when the condition of normality was not met (Perme and Manevski, 2019). The correlation between anxiety or depression or stress and Tai Chi was analyzed using the Pearson correlation coefficient test (Liu et al., 2017). Anxiety and depression were used as dependent variables, and the Tai Chi exercise was used as the independent variable. Adjusted Cox proportional hazard models were used to assess the association between the 24-form Tai Chi and clinical outcome stratified by stress, anxiety, and SF-36 including age, gender, body mass index (BMI), angina, medicine, PCI, heart attack, and family history of CHD. The test level was bilaterally *P* < 0.05 considered as statistically significant.

## Results

### Study Participant Demographics and Baseline Characteristics

**FIGURE 1:**
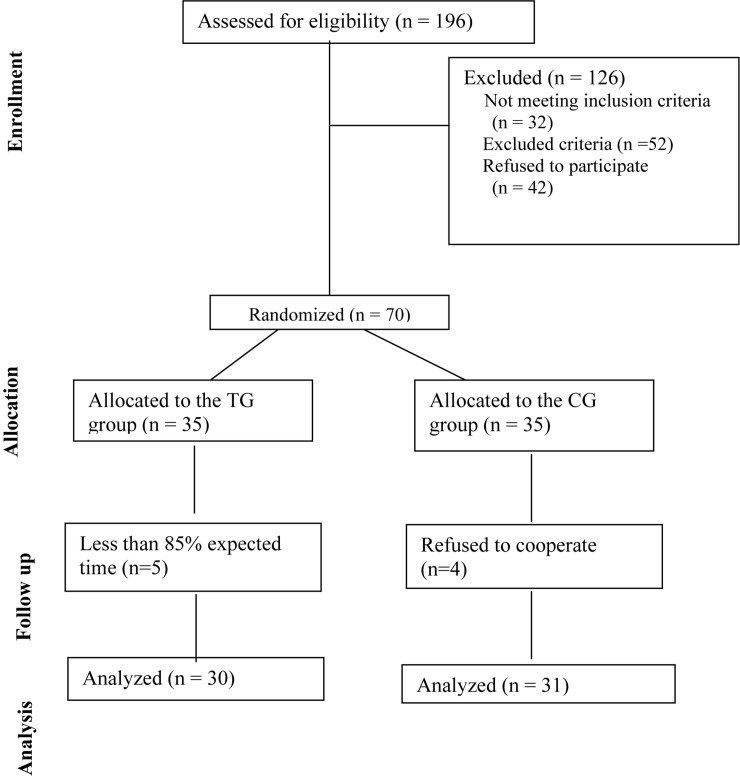
Consort diagram of the present study. The enrolled patients were numbered according to the patients’ admission order and randomly divided into the Tai Chi group (TG) and the control group (CG) by using a random number table. The duration of follow-up was 10 months.

From 1 March 2016 to 1 June 2017, 196 patients with CHD stent implantation visited the First Affiliated Hospital of Jinlin University; 112 of the patients met the inclusion criteria, 42 patients refused to participate, and eventually 70 patients were included. The patients were randomly divided into the TG and the CG groups (*n* = 35 for each group). During the trial, there were five patients who joined in the Tai Chi practice less than 80% of the expected time and recorded as “exit” in the TG group, and four patients in the CG group refused to cooperate with the unfinished indicator collection. Finally, 61 patients completed all interventions and data collection, 30 patients from the TG group and 31 patients from the CG group (Figure 1). The baseline parameters were adjusted for differences by using propensity score matching to reduce the influences of possible confounders and of collection bias. The statistical differences for the number of anxiety and depression and the stress scores were not significantly different between the two groups (Table 1, *P* > 0.05). The statistical difference was not significantly different for the baseline levels of anxiety scores, depression scores, and HADS levels, both subscale HADS-A and subscale HADS-D, between the TG and the CG groups (Table 1, *P* > 0.05). No statistical difference was observed for the other parameters between the two groups (*P* > 0.05, Table 1) after an independent sample *t*-test or χ^2^ test.

**TABLE 1:** Baseline characteristics between the two groups.

Characteristics	CG (*n* = 31)	TG (*n* = 30)	*P*-values
Age, years	56.97 ± 10.98	60.40 ± 10.86	0.225
Sex, male (%)	25 (83.33)	24 (77.42)	0.561
BMI, kg/m^2^	20.52 ± 2.46	25.19 ± 3.90	0.418
Anxiety, cases (%)	17 (54.80)	15 (50.00)	0.684
Anxiety scores	5.98 ± 3.87	6.04 ± 4.21	0.653
Depression, cases (%)	17 (54.80)	18 (60.00)	0.705
Depression scores	7.23 ± 5.46	6.91 ± 5.24	0.418
HADS	15.67 ± 5.42	16.94 ± 6.66	0.815
A	7.80 ± 3.00	8.45 ± 3.20	0.820
D	8.00 ± 3.59	8.77 ± 4.29	0.763
Stress scores	47.15 ± 6.08	47.76 ± 5.83	0.512
**Diagnosis**			
STEMI	11 (35.48)	10 (33.33)	0.860
Non-STEMI	2 (6.45)	3 (10.00)	0.969
Unstable angina	10 (32.26)	10 (33.33)	0.929
Stable angina	8 (25.81)	7 (23.33)	0.823
**PCI indication**			
Emergency PCI	10 (32.26)	8 (26.67)	0.632
Elective PCI	21 (67.74)	22 (73.33)	
Number of diseased vessels	2.00 ± 0.73	1.80 ± 0.96	0.365
Number of stents	1.42 ± 0.62	1.51 ± 0.82	0.430
Type of medicine	5.58 ± 1.57	5.47 ± 1.20	0.751
Hypertension	13 (41.94)	18 (60.00)	0.158
Diabetes	8 (25.81)	7 (23.33)	0.823
Hyperlipidemia	1 (3.23)	3 (10.00)	0.354
Family history of early-onset coronary heart disease	0 (0.00)	1 (3.33)	0.492
Heart attack	4 (12.90)	3 (10.00)	1.000
Seeking psychological counseling	0 (0.00)	0 (0.00)	NS

*Note: All data are expressed as *n*(%) or mean ± standard deviation. NS, no statistical data; BMI, body mass index; SETM, ST-segment elevation myocardial infarction; non-STEMI, non-ST-segment elevation myocardial infarction. The statistical difference was significant if *P* < 0.05.*

### Intervention Effects

#### Tai Chi Intervention Reduced the Severity of Depression and Anxiety

Tai Chi intervention time was 290–360 days, with an average time of 300.9 days. The completion rate of the intervention program was 80.56–100%, with an average of 85.93%.

**FIGURE 2:**
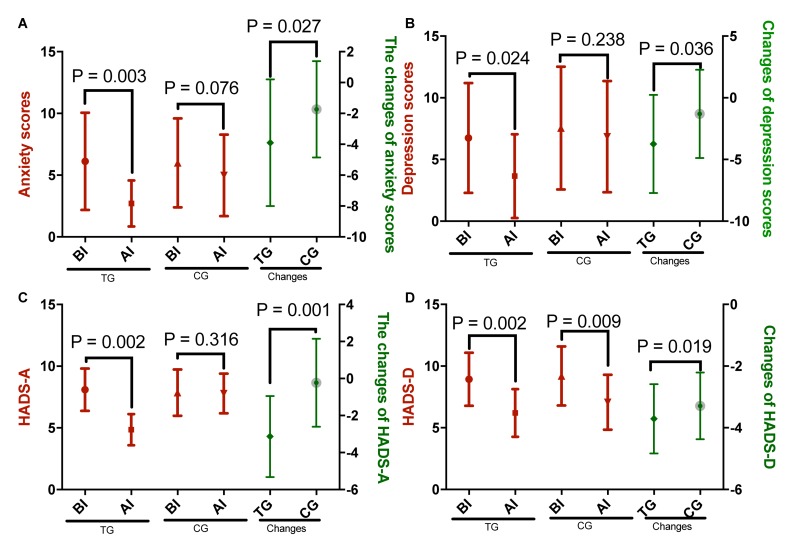
The effects of Tai Chi on anxiety and depression scores. **(A)** Anxiety scores, **(B)** depression scores, **(C)** HADS-A, and **(D)** HADS-D. The enrolled patients were numbered according to the patients’ admission order and randomly divided into the Tai Chi group (TG, *n* = 30) and the control group (CG, *n* = 31). The changes stand for the difference between the scores obtained before and after intervention for both the Tai Chi and the control groups. The duration of follow-up was 10 months. AI, after intervention; BI, before intervention. The statistical difference was significant if *P* < 0.05.

The anxiety (Figure 2A) and the depression (Figure 2B) scores and the HADS-A (Figure 2C) and the HADS-D (Figure 2D) values were significantly decreased over the study period in the TG. On the other hand, only the HADS-D values were significantly decreased in the control group (CG; Figure 2D). Changes in the anxiety and the depression scores and in the HADS-A and the HADS-D values were significantly higher in the TG when compared with those of the CG (Figure 2, *P* < 0.05). The results suggested that Tai Chi intervention improved the symptoms of anxiety and depression in the CHD patients.

#### Tai Chi Intervention Reduced the Stress of CHD Patients After PCI

**FIGURE 3:**
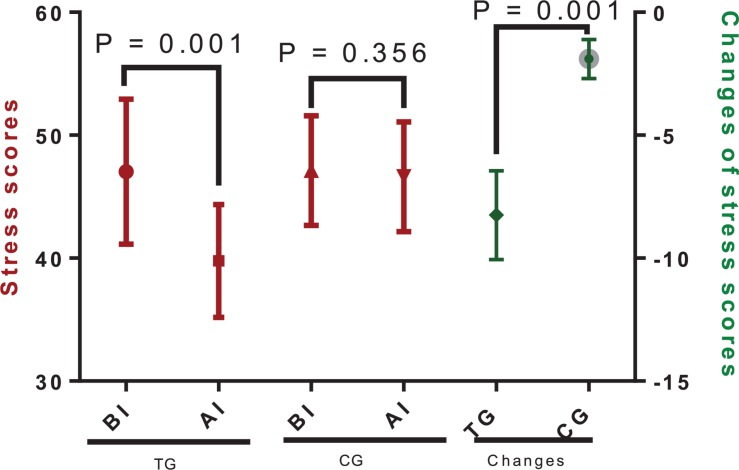
The effects of Tai Chi on stress scores. The enrolled patients were numbered according to the patients’ admission order and randomly divided into the Tai Chi group (TG, *n* = 30) and the control group (CG, *n* = 31). The changes stand for the difference between the scores obtained before and after intervention for both the Tai Chi and the control groups. The duration of follow-up was 10 months. AI, after intervention; BI, before intervention. The statistical difference was significant if *P* < 0.05.

The stress scores were significantly decreased over the study period in the TG (Figure 3, *P* = 0.001) but not in the CG (Figure 3, *P* = 0.351). Additionally, changes in the stress scores were significantly higher in the TG when compared with those of the CG (Figure 3, *P* = 0.001). The results suggested that Tai Chi intervention improved the symptoms of anxiety and depression in the CHD patients.

#### Tai Chi Intervention Improved the Life Quality of CHD Patients After PCI

The statistical difference for SF-36 scores was not significantly different between the two groups before Tai Chi intervention (*P*> 0.05, Table 2). The SF-36 scores in the TG group were higher than in the CG group after Tai Chi intervention (*P* < 0.05, Table 2). These results suggested that Tai Chi improved the life quality of CHD patients after PCI.

**TABLE 2:** The effects of Tai Chi on the life quality of CHD patients after PCI.

Variables	TG, *n* = 30		*P*-values	CG, *n* = 31		*P-*values	Change over study period		
					
	Before intervention	After intervention		Before intervention	After intervention		TG	CG	*P*-values
PF	56.89.6	86.68.5	0.001	56.19.2	72.37.2	0.001	35.125.58	17.463.69	0.011
RP	64.18.0	89.97.4	0.001	63.28.1	74.26.9	0.001	33.724.50	13.184.90	0.002
BP	72.68.2	86.88.2	0.018	72.08.5	72.57.9	0.892	16.311.75	0.980.23	0.023
SF	75.18.2	79.58.2	0.078	74.28.3	68.18.0	0.012	6.240.79	−6.151.16	0.003
VT	58.09.1	88.78.5	0.001	57.39.4	70.67.6	0.010	31.241.45	13.183.15	0.001
RE	71.39.6	79.68.1	0.039	72.48.1	64.97.2	0.005	8.471.93	−8.121.44	0.002
MH	57.38.2	85.27.9	0.001	56.48.3	70.38.3	0.001	29.341.68	14.521.19	0.001
GH	63.18.2	87.87.1	0.001	62.58.0	72.47.0	0.003	24.773.63	11.641.65	0.003

*Note: The SF-36 version 2 consists of assessing physical functioning (PF; 10 items), social functioning (SF; two items), role limitation due to physical health (RP; four items), bodily pain (BP; two items), mental health (MH; five items), role limitations due to emotional health (RE; three items), vitality (VT; four items), and general health perceptions (GH; five items) (Ware, 2000). Adjusted Cox proportional hazards models were used to assess the association between the 24-form Tai Chi and the clinical outcome stratified by depression, anxiety, and SF-36 including age, gender, BMI, angina, medicine, PCI, heart attack, and family history of CHD. The statistical difference was significant if *P* < 0.05.*

#### Tai Chi Intervention Increased the Serum Level of miR-17-92

**FIGURE 4:**
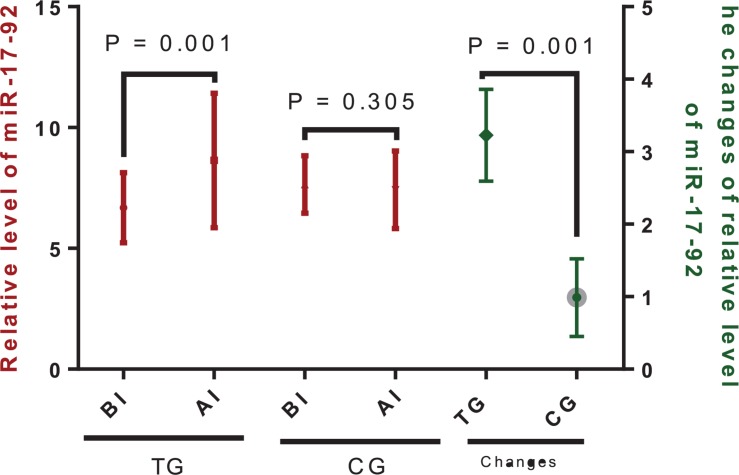
Relative levels of miR-17-92 between the Tai Chi (TG) and the control (CG) groups. The enrolled patients were numbered according to the patients’ admission order and randomly divided into the Tai Chi group (*n* = 30) and the control group (*n* = 31). The changes stand for the difference between the scores obtained before and after intervention for both the Tai Chi and the control groups. The duration of follow-up was 10 months. AI, after intervention; BI, before intervention. The statistical difference was significant if *P* < 0.05.

The relative levels of miR-17-92 were significantly increased over the study period in the TG (Figure 4, *P* = 0.001) but were not statistically different in the CG (Figure 3, *P* = 0.305). Moreover, changes in the relative levels of miR-17-92 were significantly higher in the TG when compared with those of the CG (Figure 4, *P* = 0.001). The results suggested that Tai Chi exercise increased the serum level of miR-17-92 as revealed by the change.

### Correlation Between miR-17-92 and Emotional Disorders

**FIGURE 5:**
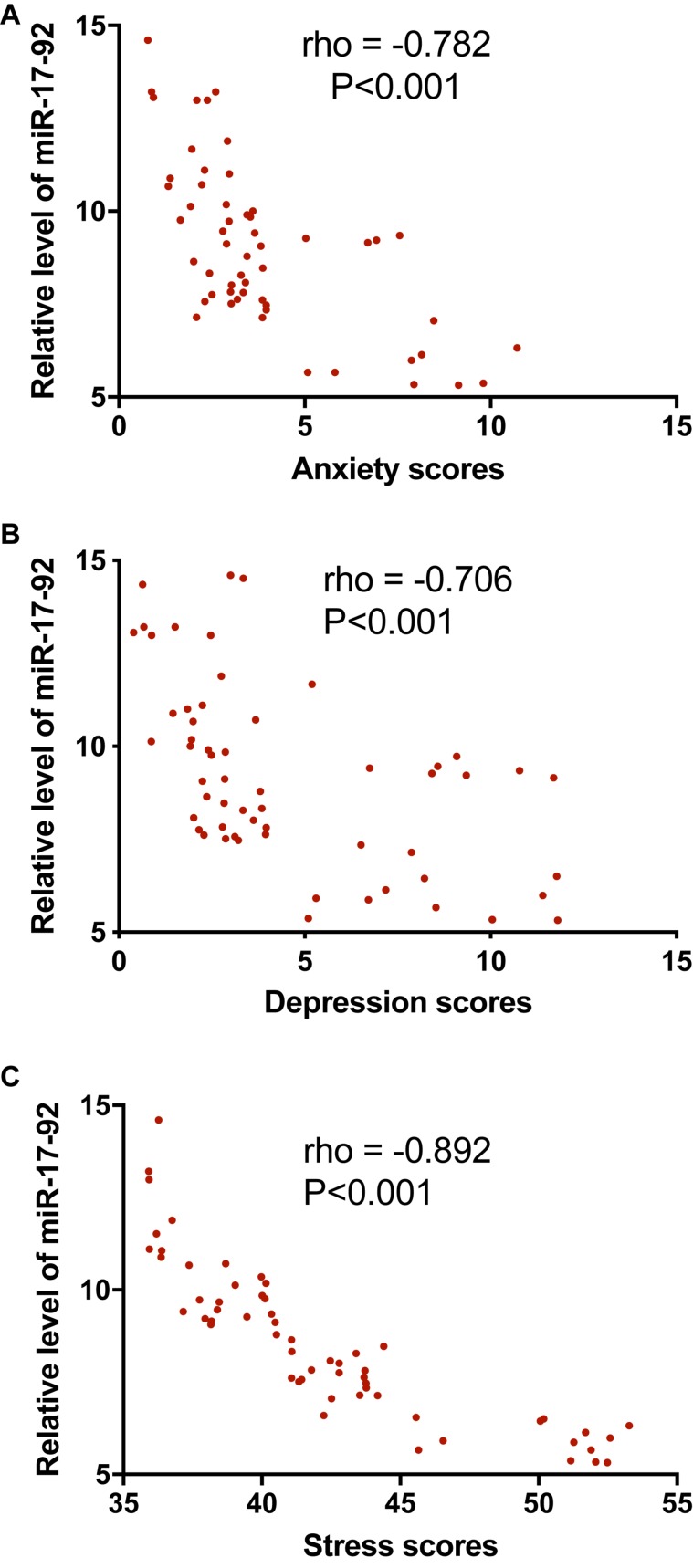
The correlation between the relative levels of miR-17-92 and anxiety, depression, and stress scores. **(A)** The correlation between the relative levels of miR-17-92 and the anxiety scores. **(B)** The correlation between the relative levels of miR-17-92 and the depression scores. **(C)** The correlation between the relative levels of miR-17-92 and the stress scores. There was a strong negative correlation between the two variables if rho values <−−0.5.

Pearson correlation coefficient test showed that, with the increase in the serum levels of miR-17-92, the levels of anxiety (Figure 5A), the scores for depression (Figure 5B), and the stress scores (Figure 5C) were reduced significantly (*P* < 0.001). The serum level of miR-17-92 had negative correlation with anxiety, depression, and stress scores since rho <−0.5 and *P* < 0.001.

## Discussion

According to the GAD-7 and the PHQ-9 score assessments, there were 17 patients with anxiety and 17 patients with depression in the CG group and 15 patients with anxiety and 18 patients with depression in the TG group before intervention (Table 1, *P* = 0.684 and 0.705, respectively). Comparatively, there were 11 patients with anxiety and 14 patients with depression in the CG group and four patients with anxiety and seven patients with depression in the TG group after intervention (*P* = 0.045 and 0.073, respectively). In a similar case, the statistical difference was not significantly different for the stress score before intervention (Table 1, *P* = 0.512), and the statistical difference was significant after intervention (*P* = 0.001). Most patients in both groups had mild anxiety and depression (according to the GAD-7 and the PHQ-9 score assessments), in which the patients experienced instability in emotionality, relationships, and behavior. The anxiety and depression symptoms were further measured by the HADS. Both evaluations showed consistency. The perceived stress was associated with a CHD incident and typical symptoms included worry and rumination. The patients further exhibited sleep problems.

This study also showed that there was no significant difference in HADS score, anxiety subscale score, depression subscale score, and the proportion of the patients with anxiety or depression and stress between the two groups before intervention. The findings demonstrated that Tai Chi improved the depression and the anxiety symptoms and reduced the pressure of self-perception in the CHD patients after PCD and was not affected by baseline characteristics.

Tai Chi exercise can significantly improve the quality of life of patients. Other studies have also confirmed that Tai Chi can effectively improve the patient’s circulation and balance, can help relax and strengthen the nervous system (Zhang, 2015a), and can lead to good regulation of the cardiovascular system (Liu et al., 2018) and the respiratory system (Song et al., 2014; Siu, 2016) to effectively improve the quality of life of the patients.

Tai Chi improved the symptoms of emotional disorders in patients with CHD after PCI. Tai Chi is combined mind and physical exercise and may be more effective than aerobic exercise in preventing some diseases (Ostrovsky, 2018).

The critical role of miRNA dysregulation in psychiatric disorders has been widely reported. For instance, preclinical evidence supports that the microRNA-34 family is involved in stress-related psychiatric conditions and in the modulation of depression (Lo Iacono et al., 2019). miR-140-5p has been found to be associated with the pathogenesis of late-onset post-stroke depression (Liang et al., 2019). The miR-101a-3p and its target, enhancer of zeste homolog 2 (Ezh2) in the amygdala, contribute to anxiety-like behavior (Cohen et al., 2017). The microRNA levels may change based on the training type or exercise (Domanska-Senderowska et al., 2019). Aerobic exercise delays neurodegenerative diseases and lesions by regulating miR-3557/324 (Liu et al., 2019). It is quite possible that Tai Chi also induces the changes in miRNA. The present work also showed that the serum level of miR-17-92 was increased after Tai Chi intervention. Furthermore, a negative correlation between the serum level of miR-17-92 and the scores of anxiety, depression, and stress was observed (Figure 5). The results suggested that the miR-17-92 changes may contribute to the emotional changes.

There were some limitations in the present study: only the levels of anxiety, depression, stress, and SF-36 were measured in the CHD patients after PCI within 1 year and repeated evaluations were not performed until 1 year later, so the persistent effects of Tai Chi on adverse cardiovascular events were not determined. “HADS” was used to assess the patients’ anxiety and depression, and psychological interviews were not conducted. The exact molecular mechanism for the functional role of Tai Chi was not explored in the CHD patients after PCI. The possible side effects of Tai Chi were not investigated, although most reports showed that Tai Chi could reduce most side effects of various diseases (Murley et al., 2019; So et al., 2019). According to our experiences, there is a difference in learning Tai Chi between males and females, but the issue was not explored in the present study. Further work is highly needed in the future.

## Conclusion

Tai Chi improved the symptoms of anxiety, depression, and stress and upregulated the miR-17-92 in CHD patients after PCI. Tai Chi also improved the quality of life of the CHD patients. This is suggestive that Tai Chi should be used as a potential way to improve the emotional parameters of the CHD patients.

## Data Availability Statement

The raw data supporting the conclusions of this article will be made available by the authors, without undue reservation, to any qualified researcher.

## Ethics Statement

The studies involving human participants were reviewed and approved by this Ethics Committee of The First Hospital of Jilin University. The patients/participants provided their written informed consent to participate in this study.

## Author Contributions

WL and XW designed the study, performed the experiment, and revised the manuscript. JL and PY performed the experiment and wrote the manuscript. All authors read and approved the final manuscript.

## Conflict of Interest

The authors declare that the research was conducted in the absence of any commercial or financial relationships that could be construed as a potential conflict of interest.

## Abbreviations

BP: bodily pain
CHD: coronary heart disease
GH: general health perceptions
HADS: Hospital Anxiety and Depression Scale
MACEs: major adverse cardiovascular events
MH: mental health
PCI: percutaneous coronary intervention
PF: physical functioning
PSS: Perceived Stress Scale
RE: role limitations due to emotional health
RP: role limitation due to physical health
SF: social functioning
SSA: subsyndromal anxiety
SSD: subsyndromal depression
VT: vitality.
